# A modified Delphi study to determine the level of consensus across the European Union on the structures, processes and desired outcomes of the management of polypharmacy in older people

**DOI:** 10.1371/journal.pone.0188348

**Published:** 2017-11-20

**Authors:** Derek Stewart, Kathrine Gibson-Smith, Katie MacLure, Alpana Mair, Albert Alonso, Carles Codina, Antonio Cittadini, Fernando Fernandez-Llimos, Glenda Fleming, Dimitra Gennimata, Ulrika Gillespie, Cathy Harrison, Ulrike Junius-Walker, Przemysław Kardas, Thomas Kempen, Moira Kinnear, Pawel Lewek, Joao Malva, Jennifer McIntosh, Claire Scullin, Birgitt Wiese

**Affiliations:** 1 School of Pharmacy and Life Sciences, Robert Gordon University, Aberdeen, Scotland, United Kingdom; 2 Effective Prescribing and Therapeutics, Health and Social Care Directorate, Scottish Government, Edinburgh, Scotland, United Kingdom; 3 Directorate of Research and Innovation, Fundació Clínic per a la Recerca Biomèdica (FCRB), Barcelona, Spain; 4 Pharmacy Department, Hospital Clínic de Barcelona, Barcelona, Spain; 5 Department of Translational Medical Sciences, University Federico II, Naples, Italy; 6 Institute for Medicines Research (iMed.Ulisboa), Department of Social Pharmacy, Faculty of Pharmacy, University of Lisbon, Lisbon, Portugal; 7 Medicines Optimisation Innovation Centre, Northern Health & Social Care Trust, Belfast, Northern Ireland; 8 Department of Social and Educational Policy, University of Peloponnese, Corinthos, Greece; 9 Pharmacy Department, Uppsala University Hospital, Uppsala, Sweden; 10 Department of Health Social Services and Public Safety, Belfast, Northern Ireland; 11 Institute for General Medicine, Medizinische Hochschule Hannover, Hannover, Germany; 12 Department of Family Medicine, Medical University of Lodz, Lodz, Poland; 13 NHS Lothian Pharmacy Service, Western General Hospital, Edinburgh, Scotland, United Kingdom; 14 Faculty of Medicine, University of Coimbra, Coimbra, Portugal; 15 School of Pharmacy, Queen’s University, Belfast, Northern Ireland, United Kingdom; University of Manitoba, CANADA

## Abstract

**Background:**

Inappropriate use of multiple medicines (inappropriate polypharmacy) is a major challenge in older people with consequences of increased prevalence and severity of adverse drug reactions and interactions, and reduced medicines adherence. The aim of this study was to determine the levels of consensus amongst key stakeholders in the European Union (EU) in relation to aspects of the management of polypharmacy in older people.

**Methods:**

Forty-six statements were developed on aspects of healthcare structures, processes and desired outcomes, with consensus defined at ≥ 80% agreement. Panel members were strategists (e.g. directors, leading clinicians and commissioners) from each of the 28 EU member states, with a target recruitment of five per member state. Three Delphi rounds were conducted via email, with panel members being provided with summative results and collated, anonymised comments at the commencement of Rounds 2 and 3.

**Results:**

Ninety panel members were recruited (64.3% of target), with high participation levels throughout the three Delphi rounds (91.1%, 83.3%, 72.2%). During Round 1, consensus was obtained for 27/46 statements (58.7%), with an additional two statements in Round 2 and none in Round 3. Consensus was obtained for statements relating to: potential gain arising from polypharmacy management (3/4 statements); strategic development (7/7); change management (5/7) indicator measures (4/6); legislation (0/3); awareness raising (5/5); polypharmacy reviews (5/7); and EU vision (0/7). Analysis of free text comments indicated that the vision statements were too ambitious and not achievable by the specified timeframe of 2025.

**Conclusion:**

Consensus was obtained amongst key EU strategists around many aspects of polypharmacy management in older people. Notably, no consensus was achieved in relation to statements relating to the need to alter legislation in areas of healthcare delivery, remuneration and practitioner scope of practice. While the vision for the EU by 2025 was considered rather ambitious, there is great potential and clear opportunity to advance polypharmacy management throughout the EU and beyond.

## Introduction

Recent years have seen marked shifts in global population demographics, with increasing life expectancy, numbers, and proportions of older people [1]. Healthy and active ageing, ‘the process of optimising opportunities for health, participation and security to enhance quality of life as people age’ [2], through the design and delivery of person-centred healthcare, is fundamental to the health policies of many countries [3,4]. This is not without its challenges given the association between age and prevalence of multimorbidity [5,6], in addition to the consequent increases in healthcare services utilisation [7], and reduced quality of life [8]. Furthermore, there is extensive global evidence of the high frequency of prescribing potentially inappropriate medicines in older, multimorbid people [9–11]. Appropriate prescribing of medicines in older people is challenging given age-related physiological changes which impact pharmacodynamics and pharmacokinetics While the plethora of evidence based guidelines for single disease states may provide support, they do not provide sufficient coverage of issues relating to multimorbidity [12].. The cumulative impact of treatment recommendations results in the prescribing of an overwhelming number of medicines [13].

Polypharmacy is a widely used term which traditionally was interpreted as the concomitant use of four or five different prescribed medicines [14,15]. This emphasis on the number of medicines has come under criticism for being arbitrary and not considering appropriateness in the individual More recently, it has been suggested to adopt the terms ‘inappropriate polypharmacy’ (prescribing of multiple medicines which are either inappropriate or no longer indicated) and ‘appropriate’ or ‘optimal polypharmacy’ (appropriate prescribing of multiple medicines) [16]. Inappropriate polypharmacy has been described as ‘one of the greatest prescribing challenges’ [13], with consequences including increased prevalence and severity of adverse drug events and drug interactions, and reduced medicines adherence [13–16].

Although the goals of managing inappropriate polypharmacy and striving for appropriate polypharmacy are paramount, there is a lack of high level evidence on optimal approaches. A systematic review of the effectiveness of interventions to improve appropriate polypharmacy and reduce medicines-related problems in older people highlighted the uncertainty of which specific elements of interventions impacted appropriate polypharmacy [16]. A more recent systematic review and meta-analysis explored the impact of strategies to reduce inappropriate polypharmacy on outcomes of mortality, hospitalisation and change in number of medicines prescribed. The authors noted that the interventions were complex and concluded that there is no convincing evidence that the strategies were effective in reducing inappropriate polypharmacy or had an impact on clinically relevant endpoints [17]. This is despite the numerous different tools developed to promote appropriate prescribing in older people, many of which lack methodological robustness and validation in clinical settings [18]. While there is a need for evidence based guidance on the management of inappropriate polypharmacy and promoting appropriate polypharmacy in older people, focus on the implementation and sustainability of systems and processes is also warranted.

SIMPATHY (Stimulating Innovation Management of Polypharmacy and Adherence in The Elderly) was a pan-European initiative, funded by the European Commission to help address the issue of inappropriate polypharmacy across the European Union (EU). SIMPATHY aims to stimulate, promote and support innovation across the EU in the management of appropriate polypharmacy in the elderly, in order to contribute to efficient and sustainable healthcare systems [14]. There is emphasis on translating evidence to practice impacting healthcare structures, processes and patient outcomes (clinical, humanistic and economic). Work packages comprise a systematic review of the published and grey literature of identified policies and guidelines; case studies of the management of polypharmacy; a benchmarking survey; and PESTEL (Political, Economic, Sociocultural, Technological, Environmental and Legal) and SWOT (Strengths, Weaknesses, Opportunities and Threats) analyses. Results have demonstrated clearly the diversity across the EU and that there is opportunity to harness expertise and experiences.

The aim of this study was to determine the levels of consensus amongst key stakeholders in the EU in relation to aspects of the management of polypharmacy in older people. Particular consideration was given to aspects of structures, processes and desired outcomes around the promotion of appropriate polypharmacy.

## Methods

### Design

A consensus-based approach, utilising a modified Delphi technique, was employed. Consensus, also referred to as ‘collective agreement’, designs are employed in a variety of circumstances, notably to develop guidelines and policies in situations of limited evidence [19]. Consensus designs are group facilitation approaches which aim to determine the level of consensus among a group of experts (stakeholders) by aggregation of opinions into refined agreed opinion. The three main consensus designs are the Delphi technique, the consensus development technique and the nominal group technique [20–22]. The Delphi technique typically requires multiple administrations of anonymised questionnaires, usually over two or three rounds [23]. The first round of data collection, in the classic Delphi technique, generates qualitative data which is then used to guide development of statements for the questionnaires rounds. A modified Delphi technique, which omits the qualitative round, can be employed in those situations where the statements are derived from the literature or previous research, as in this study. Both classic and modified Delphi techniques afford participants anonymity, thereby ensuring that dominant participants do not unduly influence data collection. The Delphi technique is further characterised by participant feedback of statistical data and comments, affording participants the opportunity to reconsider their initial responses in subsequent rounds [24,25]. Given the need to collect data across the EU, the modified Delphi technique was selected over the consensus development technique and the nominal group technique which require participants to meet face to face.

### Statement development

The statements for the first round of the Delphi were developed from the findings generated from previous work undertaken as part of the SIMPATHY project, including focus groups and discourse analysis of face to face interviews [14]. Statements focused on structures, processes and desired outcomes around the promotion of appropriate polypharmacy in older people. Draft statements were developed and reviewed by members of the project team for appropriateness and clarity.

The Round 1 questionnaire was developed in Survey Monkey (Survey Monkey Inc., San Mateo, California, USA) and functionality pilot tested by the project team.

### Determining consensus

A six-point Likert scale was used for participants to rate their level of agreement or disagreement with each statement: strongly disagree; disagree; somewhat disagree; somewhat agree; agree; and strongly agree. A variety of means of assessing the point of consensus have been outlined in the literature: a predetermined number of rounds; subjective analysis; certain level of agreement; average percent of majority opinions cut-off rate; mode, mean, median ratings and rankings; interquartile range; coefficient of variation; and post-group consensus [26]. While there is no agreement on the best approach, the ‘certain level of agreement’ is the most commonly used and hence was adopted. In addition, there is no accepted, set standard for the target percentage of agreement, and while 70% (summative of agree and strongly agree) is commonly reported in the literature, given the importance of promoting appropriate polypharmacy, the consensus was deemed to have been met at 80% (summative of agree and strongly agree) for each individual statement.

### Expert panel members

Appropriate selection of ‘expert panel members’, or participants, is critical in promoting robustness and data validity. For this Delphi, the panel were to be five members from each EU member state (n = 28), giving a total of 140 members. Furthermore, these five would represent one policy maker, two healthcare commissioners, one healthcare provider director level and one clinician (physician or pharmacist).

### Recruitment of expert panel members

The recruitment and engagement of the panel members is crucial to the success of any Delphi. Response rates are typically 85% and any reduction may compromise the internal and external validity of the findings [26]. For SIMPATHY partner countries (United Kingdom [Scotland, Northern Ireland], Italy, Germany, Greece, Poland, Portugal, Spain), one member of the project team in that country was responsible for the initial contact with potential participants and for securing agreement to participate. Project team members employed several approaches including their professional networks, those of colleagues etc. In addition, those initial contacts unable to participate were requested to nominate other like individuals. This was continued until the required number and types of panel members were recruited or until the end of the time period for recruitment. For non-partner countries, several sources including professional networks were used to identify potential panel members. These were allocated to members of the project team and a similar approach to recruiting within partner countries was adopted. Each potential panel member was provided with full study information, outlining the aim of the Delphi, the extent and timing of their expected involvement and the potential societal benefits. In addition, they were each requested to complete, and return electronically, a consent form.

### Delphi rounds

#### Round 1

At the point of commencing the Delphi in November 2016, each panel member was sent an email with a link to the online questionnaire. In addition to the Delphi statements, the online questionnaire captured the professional role and country of residence of each panel member. A two-week deadline was set for rating levels of agreement or disagreement with each statement. A comments box was included for each statement, allowing justification of responses and the opportunity to propose new statements. All panel members were sent a reminder email at weekly intervals. Data generated from completing the online questionnaire were extracted to Microsoft Excel for descriptive analysis (frequencies and percentages) to identify whether or not consensus had been obtained for each statement. A content analysis approach was performed on any textual responses [27].

#### Round 2

In Round 2, collective feedback of all panel members’ Round 1 responses was provided, highlighting levels of consensus achieved for each statement and also all comments for each statement where consensus was not achieved. The Round 2 questionnaire required participants to rate only those statements that did not meet consensus in round one. As in Round 1, a two-week deadline was given for completion and return, with the approach to reminders and analysis the same as Round 1.

#### Round 3

Round 3 proceeded as per Round 2. Those statements not achieving consensus in Round 3 were deemed non-agreement.

## Results

### Expert panel member recruitment

Ninety-five expert panel members were recruited, with four EU member states over-recruiting, giving an adjusted recruitment rate of 90/140, 64.3%. The target of five experts per member state was achieved in 13/28 states, 46.4%, with no recruitment in six (21.4%) member states (Croatia, Estonia, France, Latvia, Lithuania, Luxembourg).

### Expert panel member participation

The participation of panel members is given in Table 1, with rates of 91.1%, 83.3% and 72.2% achieved in Rounds 1, 2 and 3 respectively. The target of five members per EU member state was achieved in eight states in Round 1, five in Round 2 and three in Round 3.

**Table 1:** Panel member participation in each of the three Delphi rounds.

Country	Round 1	Round 2	Round 3
Austria	4	4	4
Belgium	3	3	3
Bulgaria	1	0	0
Croatia	0	0	0
Cyprus	3	3	3
Czech Republic	3	3	3
Denmark	5	4	3
Estonia	0	0	0
Finland	3	4	4
France	0	0	0
Germany	5	5	5
Greece	5	5	4
Hungary	5	5	3
Ireland	4	4	3
Italy	2	1	1
Latvia	0	0	0
Lithuania	0	0	0
Luxembourg	0	0	0
Malta	2	2	2
Netherlands	5	3	2
Poland	5	4	4
Portugal	4	5	2
Romania	3	1	2
Slovakia	2	1	1
Slovenia	5	5	5
Spain	4	4	3
Sweden	4	4	3
United Kingdom	5	5	5
**Total participation**	**82/90 (91.1%)**	**75/90 (83.3%)**	**65/90 (72.2%)**

Panel member roles are given in Table 2, with many having multiple roles. The recruitment of commissioners was particularly challenging.

**Table 2:** Professional roles of Delphi panel members.

Roles	Round 1 (n = 82)	Round 2 (n = 75)	Round 3 (n = 65)
Lead/ Director/ Head/ Chief/ Chair	30	27	19
Physician	29	29	21
Pharmacist	28	28	23
Academic	22	19	18
Commissioner	5	2	4
Politician	1	1	0
Nurse	1	0	0
Patient organisation	1	1	0

### Statement consensus

Consensus was obtained for 27/46 (58.7%) statements in Round 1, with consensus obtained for a further two statements (29/46, 63.0%) in Round 2 and nil in Round 3. Tables 3–8 illustrate those statements for which consensus was obtained, presented according to each of the distinct sections. Consensus was obtained as follows for statements relating to: potential gain arising from polypharmacy management (3/4 statements); strategic development (7/7); strategic change management (5/7); indicator measures (4/6); legislation (0/3); awareness raising (5/5); polypharmacy reviews (5/7); and EU vision (0/7).

**Table 3:** Number of responses and % agreement (strongly agree/agree) to statements relating to potential gain arising from polypharmacy management.

Statements	SD	D	So D	So A	A	SA	% A
There is a need for an EU level coordinated approach to identify, share, disseminate, promote and support best practice around polypharmacy management (n = 82, Round 1)	0	2	1	3	36	40	93
Polypharmacy management should lead to considerable health gain (n = 82, Round 1)	0	1	0	7	33	41	90
Polypharmacy management should lead to better healthcare workforce utilisation and efficiency (n = 82, Round 1)	0	0	3	8	44	27	87

(SD = strongly disagree, D = disagree, So D = somewhat disagree, So A = somewhat agree, A = agree, SA = strongly agree, %A = % agreement)

**Table 4:** Number of responses and % agreement (strongly agree/agree) to statements relating to strategic development.

Statements	SD	D	So D	So A	A	SA	% A
Leaders of polypharmacy management should articulate a clear vision encompassing aims, objectives, motivating factors and outcomes (n = 82, Round 1)	0	0	0	3	37	42	96
Leaders of polypharmacy management should ensure that the strategic vision is shared and understood by all involved in implementation (n = 82, Round 1)	0	0	0	4	28	50	95
Polypharmacy management should be overseen by a diverse range of stakeholders including policy makers, physicians, pharmacists, nurses, and patients or patient advocates (n = 82, Round 1)	1	0	3	6	28	44	88
Polypharmacy management should be incorporated into health policy strategies at local, regional and national levels that guide the course of care delivery (n = 82, Round 1)	0	0	0	5	34	43	94
Polypharmacy management should be evaluated fully prior to large scale implementation, encompassing both quantitative outcome measures and the perspectives of key stakeholder groups (n = 81, Round 1)	1	2	5	9	35	39	79*
Information and communications technology tools should be developed and implemented across all healthcare settings to support polypharmacy management (n = 81, Round 1)	0	0	1	3	31	46	95
Polypharmacy management should be developed as essential components of larger initiatives in the healthcare system such as patient safety, management of long term conditions, and care for older people (n = 82, Round 1)	0	1	0	3	28	50	95

(SD = strongly disagree, D = disagree, So D = somewhat disagree, So A = somewhat agree, A = agree, SA = strongly agree, %A = % agreement)

* statements with 79% agreement were so close to the 80% cut-off that they were also deemed as achieving consensus

**Table 5:** Number of responses and % agreement (strongly agree/agree) to statements relating to change management.

Statements	SD	D	So D	So A	A	SA	% A
When developing polypharmacy management, leaders should develop an explicit change management strategy and plan (n = 82, Round 1)	0	0	5	12	39	26	79*
Prior to implementation of polypharmacy management, the culture of the organisation should be assessed for both strengths and potential barriers to implementation (n = 82, Round 1)	0	3	2	6	33	28	87
A detailed assessment of the need for additional resources required to support the implementation and evaluation of polypharmacy management should be undertaken (n = 82, Round 1)	0	4	2	11	36	29	79*
Leaders of polypharmacy management should work across care settings and boundaries to ensure implementation is in a standard manner throughout the health system (n = 82, Round 1)	1	1	2	13	28	37	79*
The development of the clinical pharmacy workforce, particularly in primary care, will be a key enabler of service provision around polypharmacy management (n = 75, Round 2)	1	0	4	7	26	37	84

(SD = strongly disagree, D = disagree, So D = somewhat disagree, So A = somewhat agree, A = agree, SA = strongly agree, %A = % agreement)

* statements with 79% agreement were so close to the 80% cut-off that they were also deemed as achieving consensus

**Table 6:** Number of responses and % agreement (strongly agree/agree) to statements relating to indicator measures.

Statements	SD	D	So D	So A	A	SA	% A
There is a need to develop valid, reliable and sensitive indicators to quantify the extent of inappropriate and appropriate polypharmacy (n = 82, Round 1)	1	1	3	4	29	44	89
There is a need to develop valid, reliable and sensitive indicators to quantify the impact on patient clinical outcomes relating to the extent of inappropriate and appropriate polypharmacy (n = 82, Round 1)	0	1	1	7	21	52	89
Valid, reliable and sensitive process indicators relating to polypharmacy management should be developed (n = 82, Round 1)	0	1	1	10	32	38	85
Data relating to valid, reliable and sensitive indicators of inappropriate and appropriate polypharmacy should be reported routinely to key stakeholder groups at local, regional and national levels (n = 75, Round 2)	0	0	3	7	30	34	85

(SD = strongly disagree, D = disagree, So D = somewhat disagree, So A = somewhat agree, A = agree, SA = strongly agree, %A = % agreement)

**Table 7:** Number of responses and % agreement (strongly agree/agree) to statements relating to awareness raising.

Statements	SD	D	So D	So A	A	SA	% A
There is a need to increase awareness of the issues relating to polypharmacy amongst health policy leaders (n = 82, Round 1)	0	0	2	5	27	48	92
There is a need to increase awareness of the issues relating to polypharmacy amongst health professional leaders (n = 82, Round 1)	0	0	3	9	24	46	85
There is a need to increase awareness of the issues relating to polypharmacy amongst patient representative leaders (n = 82, Round 1)	0	0	0	11	24	47	87
Education on polypharmacy management needs to be integrated into undergraduate curricula for all health professionals, particularly doctors, pharmacists and nurses (n = 82, Round 1)	0	1	0	7	11	63	90
Polypharmacy management needs to be integrated into continuing professional development programmes for all health professionals, particularly doctors, pharmacists and nurses (n = 82, Round 1)	0	0	0	5	21	56	94

(SD = strongly disagree, D = disagree, So D = somewhat disagree, So A = somewhat agree, A = agree, SA = strongly agree, %A = % agreement)

**Table 8:** Number of responses and % agreement (strongly agree/agree) to statements relating to patient centred polypharmacy reviews.

Statements	SD	D	So D	So A	A	SA	% A
There is a need for evidence based guidelines promoting a systematic approach to patient centred polypharmacy reviews (n = 82, Round 1)	0	0	1	12	23	46	84
The roles and responsibilities of the members of the multidisciplinary team delivering patient centred polypharmacy reviews must be clearly defined and articulated (n = 82, Round 1)	0	0	1	9	33	39	88
Clinical decision support systems should be developed and implemented to facilitate patient centred polypharmacy reviews (n = 82, Round 1)	0	0	0	9	31	42	89
Those involved in delivery of patient centred polypharmacy reviews should have electronic access to all relevant patient information (n = 82, Round 1)	0	0	0	6	24	52	93
All affected patients, regardless of setting or environment, should have access to a polypharmacy review (n = 82, Round 1)	0	1	4	10	24	43	82

(SD = strongly disagree, D = disagree, So D = somewhat disagree, So A = somewhat agree, A = agree, SA = strongly agree, %A = % agreement)

### Statement non-consensus

Following three rounds, consensus was not obtained for 17 statements. Levels of agreement for each statement are provided in Table 9.

**Table 9:** Statements for which consensus was not obtained.

Statements relating to…		% Agreement		
		Round 1, n = 82	Round 2, n = 75	Round 3, n = 65
… potential gain arising from polypharmacy management	Polypharmacy management should lead to considerable economic societal gain	72	65	66
… strategic change management	Health systems should design payment mechanisms and incentives that align with the work required to implement polypharmacy management	71	67	69
	To facilitate implementation, polypharmacy management should be captured within contractual arrangements for health professionals	59	43	40
…indicator measures	There is a need to develop valid, reliable and sensitive indicators to quantify the economic impact relating to the extent of inappropriate and appropriate polypharmacy	72	73	72
	Valid, reliable and sensitive indicators of inappropriate and appropriate polypharmacy should be developed at the EU level	59	48	43
…legislation	Legislation governing healthcare delivery that limits the implementation of polypharmacy management should be addressed as part of a long-term implementation plan	63	55	66
	Legislation governing remuneration for healthcare services that limits the implementation of polypharmacy management should be addressed as part of a long-term implementation plan	60	63	60
	Legislation governing the scope of practice of relevant health professionals (e.g. nurses, pharmacists) that limits the implementation of polypharmacy management should be addressed as part of a long-term implementation plan	67	62	68
…patient centred polypharmacy reviews	There is a need for common EU evidence based guidelines promoting a systematic approach to patient centred polypharmacy reviews	66	71	69
	Each EU member state should develop their own national or regional evidence based guidelines promoting a systematic approach to patient centred polypharmacy reviews	45	43	65
…the vision for the EU by 2025	By 2025, European healthcare will be recognised widely for effective policies on the management of polypharmacy through multidisciplinary teams	57	55	52
	By 2025, innovative, coordinated and comprehensive interventions will be in place across Europe, in all settings, supporting patient empowerment, safety and addressing polypharmacy management	56	37	37
	By 2025, integrated, user friendly dedicated information and communications technology tools will be supporting the management of polypharmacy	65	64	65
	By 2025, information and communications technology systems in all healthcare organisations will be facilitating improved communication between all healthcare providers	63	67	65
	By 2025, each citizen will have a personalised healthcare record incorporating full medicines information	57	63	65
	By 2025, patient involvement and empowerment will be a key priority in all healthcare related developments	74	75	77
	By 2025, there will be a 50% reduction (compared to the current level) in patients receiving inappropriate polypharmacy	45	32	25

Many panel members provided detailed comments to explain their responses. Content analysis of these in relation to statements where consensus was not achieved identified several key, recurring themes.

Many commented on the lack of evidence that polypharmacy management should lead to considerable economic societal gain,

‘*It might even be more expensive, e.g. interventions are expensive and up to now it has not been shown that polypharmacy management is effective*.’

One commented that economic gain was not a key priority,

‘*Economic outcomes are not the main goal. Our priority and main goals are the patient safety and efficacy, and efficiency*.’

The theme of high quality care also emerged in relation to statements on payment mechanisms and incentives, contractual arrangements and legislation,

‘*I think, that implementing polypharmacy management should be somehow the concern of health professionals, doctors, nurses, pharmacists and it shouldn't be connected with additional gratification*’,

‘*Doctors and pharmacists don't need legislation governing remuneration for their healthcare services*.’

There were mixed opinions around the need for economic related indicators of polypharmacy management. While some appeared keen to support indicator development,

‘*Quantification of economic impact could generate resources for the deployment of more health care professionals*’,

*‘Health services and polypharmacy have a cost*, *thus, economic evaluation is mandatory with well-defined indicators’*,

others viewed these as less important,

‘*For me, on a personal level, this is less important than the clinical outcomes. Yet, without any sound financial backing or background, national stakeholders will probably not want to provide financial support. But even then, I think (and I cannot base this on any evidence whatsoever) that the increased incidence of clinical outcomes is the main driver for increased costs*.’

There were also diverse comments around the need for indicators to be developed at the EU level. While some considered this to be of benefit in terms of enabling change,

‘*Comparison is one of the drivers to change; also it would be interesting to see the outcome of such consensus*’,

others noted the likely difficulty of obtaining agreement,

‘*Very difficult to get consensus in this topic. Each country is different. Health care models are different too*.’

A similar theme emerged in terms of common EU evidence based guidelines around polypharmacy management,

‘*Yes, this will give the opportunity for all EU member countries to work together and provide better guidelines*’

‘*Not all EU countries have the potential, experience and resources to develop their own national or regional evidence based guidelines. Sharing information, experience, and effective collaboration could be cost effective*.’

Others, however, were more sceptical that an EU approach was likely to be effective and preferred a national approach,

‘*More focus on national context based patient centred polypharmacy reviews (e.g. integrated care, with collaboration of GPs) is better than to put energy in EU based guideline development*.’

One suggested combining EU and national approaches,

‘*There is no need for doing the same work in each country, it would be better with an EU guideline, that can be adopted according to the local setting*.’

Consensus was not achieved for any of the SIMPATHY vision statements. The overwhelming theme was that while these were aspirational, they were not considered achievable within the timescale,

‘*A fine ambition, but I don’t think this will happen*’

‘*We have been working for almost 5 years on this specific subject; there are still too many unknowns*’

‘*I am not sure about the exact year, if this is possible in 8 years….!!! In my opinion policy change needs also a change in legislation and practising and 8 years´ period is really short for both changes (in the whole EU)*’

‘*That would be great, but I'm not sure if possible in this period of time. I think more like 2030–40*.’

## Discussion

### Statement of key findings

During the course of the three Delphi rounds, consensus was achieved for 29 statements (63.0%). Key areas of consensus were around potential gain arising from polypharmacy management, strategic development, strategic change management, indicator measures, awareness raising and patient centred polypharmacy reviews. The main areas of non-consensus were in relation to legislation and the SIMPATHY vision for polypharmacy management in the EU by 2025. Panel members felt generally that there was no need for changes in legislation to support aspects of polypharmacy management. For the SIMPATHY vision for the EU by 2025, analysis of the comments provided by panel members indicated that these statements were considered too ambitious and not achievable within the timeframe of 2025.

### Strengths and weaknesses

Best practice in conducting consensus studies was followed throughout this modified Delphi study. A high level of engagement was achieved across the EU, and while this decreased as the rounds progressed, the lowest response rate was 72.2%. Many, and often detailed, comments were received from panel members to justify their responses. The panel members represented a spectrum of director level physicians, pharmacists, nurses, academics and patient representatives.

There are, however, some weaknesses to the Delphi study hence the findings should be interpreted with caution. Despite all efforts, the target to recruit five panel members per EU member state was only achieved in 11 member states, with no recruitment in six member states. Furthermore, in Round 1, the target of five responses per member state was only achieved in eight member states, with this falling to six in Round 2 and three in Round 3. Very few commissioners or politicians were recruited, with most panel members being director level health professionals. Given the key roles of commissioners and politicians in resource allocation and leading change, the lack of involvement is a weakness which may be due to the busy work schedules of these individuals. Recruitment and response biases may have been present, with those recruited and participating being those most positive. These weaknesses may have resulted in skewed finding, potentially limiting the generalisability of the results to the entire EU and beyond.

### Interpretation of findings

The appropriate use of medicines to achieve optimal health outcomes and quality of life forms one key element of the global drive to promote active and healthy ageing [2]. Given the prevalence of multimorbidities in older people and the extent of inappropriate polypharmacy [5,6,9–11], there is a need for urgent action to ensure effective and efficient deployment of healthcare structures and processes within the context of person-centred care. There is an opportunity for EU member states, organisations and associations to harness the expertise in this area, to define best practice, learn from each other and agree key targets and associated action plans. Given the extent of engagement across the EU, the findings of this consensus study may facilitate such developments within the EU and beyond. Notably, almost all panel members (93%) agreed that a co-ordinated approach at the EU level was warranted to identify, share, disseminate, promote and support polypharmacy management. While this will take considerable effort to operationalise, the World Health Organization has recently launched a global initiative aiming to reduce the level of severe, avoidable harm related to medications by 50% over the next five years [28]. Within this initiative, polypharmacy is one of the three key areas where countries and key stakeholders are asked to make strong commitments and to take action. There is also a need to better generate the evidence and disseminate findings from any national initiatives. Related SIMPATHY research identified that of the many regional or national guidance documents on the management of polypharmacy in older people, there was only one peer reviewed publication which described the development of the guidance and none which reported impact [14].

Although there were high levels of consensus around potential health gain (90%) and healthcare utilisation, and efficiency (87%), which could result from optimal polypharmacy management, the research literature in this field is lacking, as evidenced by the conclusions of two recent systematic reviews [16,17]. Consensus was not obtained around economic societal gain, comments highlighted the lack of evidence. A very recent systematic review of economic gain arising from medication reviews highlighted that most studies were of poor quality, had been conducted only in hospital settings and that limited parameters were included in the economic evaluation [29].

Consensus was achieved for all seven statements based on strategic development in terms of the need for a clearly articulated vision (96%), which is shared widely and understood at all levels including patients and their advocates (95%). The goals and aspirations of patient engagement and involvement in healthcare planning, intervention design, development and evaluation are key priorities globally [3,4]. There were also high levels of agreement that polypharmacy management should be integrated within healthcare policies at all levels (94%) and that it should be integrated within initiatives such as the management of long term conditions (95%). Integrated care is a rapidly developing field, evidenced by the introduction of the International Journal of Integrated Care in 2000, followed by the establishment of the International Network of Integrated Care (now Foundation) in 2004 [30]. While there are many definitions of integrated care), a primary focus is addressing fragmented healthcare to enable better coordinated and continuous care that improves patient experience and achieves greater efficiency and value [31]. There have been calls to include pharmacists in integrated care teams [32], and a recent meta-analysis showed that integrating pharmacists into these teams improved therapeutic, safety, hospitalization, and adherence outcomes [33].

All statements on strategic change management achieved high levels of consensus except those relating to payments and contractual arrangement for health professionals, with several commenting that there was no need for such payments or changes to contracts. Similar responses were received around the need for legislative change to promote polypharmacy management. Statements on change management were based on findings of the SIMPATHY case studies in which facilitators and barriers of change were identified using Kotter’s 8-Step Change Model [34]. One key area of consensus was around assessing organisational culture to identify enablers and barriers to change (87%). There are many different definitions of organisation culture such as ‘the pattern of basic assumptions that a given group has invented, discovered, or developed in learning to cope with its problems of external adaptation and internal integration…’ [35].. This encapsulates aspects such as values, learning, perceptions, beliefs and practices at all levels of an organisation. Acknowledging that the objective measurement of these aspects and hence culture are notoriously complex and fraught with limitations, there is evidence that within the field of patient safety, interventions can improve safety culture, potentially reducing patient harm [36]. Focusing more on the culture of the organisation may enhance the implementation of interventions around polypharmacy management.

Generating high levels of quantitative evidence of impact of change requires the application of valid, reliable and sensitive outcome measures and indeed there were high levels of consensus around the need to develop these (89%). A systematic review of the outcomes reported in trials of medication review in older patients noted the need for a standardised core outcome set to improve outcome reporting and evidence synthesis [37]. A recent consensus study of 19 Delphi panellists aimed to develop such a core outcome set for effectiveness trials aimed at optimising prescribing in older adults in care homes. Thirteen outcomes (organised into seven overarching domains: medication appropriateness, adverse drug events, prescribing errors, falls, quality of life, all-cause mortality and admissions to hospital (and associated costs)) met the criteria for inclusion in the final set [38]. While these outcomes are orientated towards care homes, they may also be of use in other settings. The current study points to the need to develop similar outcomes relating to process indicators of polypharmacy management. No consensus was obtained around the need for economic indicators or that indicators should be developed at the EU level, which is surprising given that the processes of polypharmacy management are likely to be similar.

Consensus was also achieved for most statements relating to the processes of patient centred medication reviews in the areas of need for evidence based guidelines (84%), defining the roles and responsibilities of healthcare professionals (88%) and the need for clinical decision support systems (89%). Interestingly, there was no agreement over the need for common EU evidence based guidelines or each EU member state developing their own guidelines. There is an apparent dilemma between the efficiency gained in common guidelines and the lack of ownership and possible contextualisation. It may be that there are common evidence based approaches to the process of medication reviews. This may include the use of many of the explicit and implicit measures of prescribing appropriateness [18] and issues such as the allocation of tasks to specific health professionals which may depend on issues such as competence. For example, while independent prescribing by pharmacists and nurses is widespread in the United Kingdom [39], this is not within the legislative framework of many EU member states.

The statements which had the lowest levels of consensus were those around the SIMPATHY vision for polypharmacy management in the EU by 2025 and indeed consensus was not achieved for any of these statements. A vision statement is defined as ‘a declaration of an organisation's objectives, ideally based on economic foresight, intended to guide its internal decision-making’ [40]. Analysis of comments indicated that while these were considered aspirational, they were not achievable within the proposed timeframe of 2025. It may be that members of the SIMPATHY project viewed the vision statement as aspirational and as a tool to create a sense of urgency, whereas the Delphi participants considered it on more practical terms.

### Further work

It is acknowledged that consensus based approaches generate a lower level of evidence than can be derived from experimental designs such as randomised controlled trials. Robust research is therefore warranted to progress from the Delphi study to further the development, implementation and evaluation of polypharmacy management on an international level. The United Kingdom Medical Research Council Framework relating to complex interventions could form the basis for such developments in terms of refining and further developing guidance [41]. This is then followed by phases of feasibility and pilot testing prior to wide scale evaluation incorporating objective indicators of processes and outcomes together with nested qualitative research exploring processes from multiple perspectives.

### Conclusions

In conclusion, consensus was obtained amongst key stakeholders in the EU in relation to many aspects of the management of polypharmacy in older people. Key areas of consensus were around potential gain arising from polypharmacy management, strategic development, strategic change management, indicator measures, awareness raising and patient centred polypharmacy reviews. Notably, no consensus was achieved in relation to statements relating to the need to alter legislation in areas of healthcare delivery, remuneration and practitioner scope of practice. While the SIMPATHY vision for the EU by 2025 was considered rather ambitious, there is great potential and clear opportunity to advance polypharmacy management throughout the EU and beyond.
